# Common Errors in Diagnosis. Sprains and Fractures
*A Lecture delivered at the Polyclinic.


**Published:** 1913-12-06

**Authors:** Albert Carless


### COMMON ERRORS IN DIAGNOSIS.

### Sprains and Fractures.*

By ALBERT CARLESS, M.S., F.R.C.S.

There are many articular conditions in which fractures may be mistaken for sprains or *vice versa*.

The results of such errors are often difficult to overcome. When graver injuries in the neighbourhood of joints have been overlooked by the friends, one too often hears the remark, "We thought it was only a sprain." Sometimes the practitioner has overlooked the condition, perhaps from lack of time, so that there has not been a thorough examination. But often there is a want of knowledge concerning the anatomy and physiology of joints. Teachers of anatomy are more occupied with pointing out the why and the wherefore of particular structures than the exact relationship of these parts and their importance in surgical practice. Hence, there are not a few who have a very poor idea of the physiology of joints and their normal range of movement in various directions. This range of movement varies much with the age and occupation of the person. For example, the power of abduction of the hip joint is greater in the young than in the old, so that young persons can, with training, perform what is called "the splits" without injury.

Another cause of error is the presence of excessive swelling in the neighbourhood of the joint, and the engorgement with blood which makes digital examination practically impossible. *X*-ray examination will often afford a way out in a doubtful case, but this should be done in two directions. Sometimes there is a difficulty in the interpretation of an *x*-ray plate, especially in the case of children, whose epiphyses have not yet ossified.

Where a fracture through the articular end of bone has been overlooked, it is sometimes possible for displacement of one of the fragments to occur at a later date, slight at first, but gradually increasing until the normal mechanism of the joint is hopelessly deranged; and the ultimate result is a mechanically produced arthritis. In other cases there may be persistent synovitis, due to the irritation caused by movements of bone in an ununited fracture, or by the irritation produced by adhesions and by exposure of the fractured surface of the joint.

Another trouble to bear in mind is the lighting up of either tubercular disease in children or arthritis deformans in older patients, as the result of overlooking a so-called sprain, but which in the former is really an injury to the juxta-epiphyseal bony region.

The term "sprain" should be limited to a condition in which there is some tearing of ligamentous tissues. Mere abnormal stretching is a strain. A sprain, whether applied to ligaments, muscles, or tendons, always involves the idea of rupture of tissues. There are two categories of sprain, according to whether the synovial membrane is involved or not. In the former, the joint becomes filled with blood, and the possibility of trouble arising from adhesions is multiplied. When that membrane is not involved the condition usually clears up without complications.

With regard to the diagnosis of sprain from fracture of the articular end of bone, some fractures in this region are so obvious, owing to displacement and the mobility of the fragments, with crepitus, that he who runs may read. But if there be a partial fracture without displacement, because the periosteum is not completely torn through, mistakes may easily arise. The history of the accident is of but little value: the pain due to the breaking of a bone may be no more acute than that following the rupture of a ligament.

Deformity is uncommon following pure sprain, and can scarcely occur apart from dislocation of the bone. But slight degrees of deformity are common in fracture of articular ends of bones. Therein lies a means of diagnosis which is often plain to the skilled finger. This is, of course, specially important in cases of injury to the wrist.

A slight sprain may be so tender as to render the least movement impossible. There is risk of error in regard to crepitus. The effusion of blood into a joint may cause a soft crepitus; but true bony crepitus may occur from the rubbing of exposed ends of bone together. Care must be taken in the examination of injuries to joints in the working-class whose occupations have exposed them to such strains as may determine a mechanical arthritis, such as coal-heavers. The crepitus here may be produced by the fracture of osteophytic masses.

There are other conditions to be kept in mind in particular joints in examining for supposed sprain; firstly, all the troubles connected with inter-articular cartilages. And a second condition is the breaking off of a portion of articular cartilage, or of some osteophyte.

Sprains in the region of the wrist joint are particularly common, especially as the hands are always put out for protection when falling; hence, most injuries of the wrist occur as the result of hyper-extension. There are a number of conditions in the shape of fractures and dislocations which must be excluded before the diagnosis "Only a sprain" is made. The characteristic features of a Colles's fracture may be indicated as follows:—

(1) The lower fragment of the radius is displaced backwards, so that the hand is abducted, and the back of the wrist is unduly prominent.

(2) The normal relation of the styloid processes is altered. The normal styloid process of the radius should be lower than that of the ulna. Displacement of the lower fragment of the radius necessarily brings the styloid process on to a level with the other, and this can generally be easily made out.

(3) The wrist joint itself is freely movable and without pain if care be taken to immobilise the

* A Lecture delivered at the Polyclinic.

fragments. In a sprained wrist the movements are practically always painful. This only holds good when the fracture does not involve the styloid process of the ulna. If that be broken off all movements of the wrist joint will be painful.

(4) The hand itself is held with the fingers and the metacarpal region thrown backwards, whilst there is a well-marked projection in front just above the wrist, constituting the so-called dinner-fork deformity. Oblique or longitudinal fractures of the lower end of the radius close to the styloid process are fairly obvious if carefully looked for.

In sprains of the wrist the pain is mainly localised over the lateral ligaments, and not so low down as it would be if there were a fracture through the neck of the scaphoid. Fracture of the scaphoid produces swelling in the carpal region, with considerable loss of power, and pain chiefly on the outer side of the wrist, under the region known as the anatomical snuff-box, *i.e.*, distal and internal to the styloid process of the radius. It is difficult to detect crepitus; and displacement even of the proximal fragment is uncommon. But a skiagram makes it obvious if it is taken in the right direction.

Another carpal injury is dislocation of the semilunar bone, which is characterised by a bony projection beneath the flexor tendons in the palm, with marked pain and loss of power in the wrist. Careful comparative palpation of the two wrists in the antero-posterior direction will demonstrate the existence of thickening, and there will sometimes be neuralgia of the terminal fibres of the median nerve. It may be combined with fracture of the scaphoid, and then the os magnum will be liable to project posteriorly.

The elbow is a stable joint, due to interlocking of the trochlear surface with the olecranon, so that one is not surprised to find that fractures and dislocations are more common than simple sprains, particularly in children. Much can be ascertained by simple inspection and comparison with the opposite side; and gentle examination of active and passive movements must be made, including pronation and supination. The relative lengths of arm and forearm should also be noted, and whether or not the bony points are normal.

Numerous injuries at the elbow joint are likely to be overlooked. One is partial separation of the epiphyses in children; another is fracture of the internal epicondyle. Sprains of the shoulder joint are not very common. Inspection will generally show if there is a grave lesion of the dislocation type, but the normal curve of the shoulder can be stimulated by a hæmatoma, though this can be determined by pressing with the finger below the acromion.

In the ankle joint sprains are very common, with particular liability to affect the lateral ligaments. Where there is not very obvious deformity, there are many possibilities of mistake. The history of the accident is helpful, because there is considerable modification of the type of injury according to whether the foot is abducted or adducted at the time of the accident. There are comparatively few accidents due to pure abduction; in most cases the foot is also rotated, and this makes a difference in the character of the fracture. In a sprain the joint becomes filled with blood and effusion, and the pain and tension caused thereby persist for some days, even though the limb has been kept at rest during that time. In a fracture severe pain at the time of the injury may be followed by some swelling of the part, but the pain gets less as the part is kept at rest, because the blood finds its way through the line of fracture into the surrounding tissues. Any movement, however, will light up the pain again.

Sprains of the knee joint are very common, and there need be small wonder at this when one looks at the articular surfaces of the tibia and femur and sees how little suited they appear to be for the production of a stable articulation which has to be exposed to great strain. Such a desideratum can only be secured by the development of very powerful ligaments. The stability of the joint depends principally on the arrangement of the crucial ligaments and of the posterior ligament of Winslow, and one cannot but marvel continually at the wonderful mechanical skill represented in these structures. The ligamentous structures on the outer side of the knee are more powerful than those on the inner, and so sprains on that side are less common.

Note the condition brought about when the femur and tibia are articulated together. The interval between the bones is only partially filled by semilunar cartilages, and pads of fat must be introduced to fill up the space. These pads are liable to injury and inflammation, and derangements of the knee joint following strain or injury may be induced by hæmorrhage into or bruising of these structures.

It is easy to recognise the existence of effusion into a knee joint after an injury, whether that injury be contusion or sprain. The tension of the fluid lifts the patella from the intercondyloid part of the femur, and riding of the bone can be detected. The appearance and shape of the articulation are very characteristic. Contusion of the joint usually results from injury applied directly to it, and there will be bruises on the skin, with a local tenderness which is not necessarily most marked over the lateral ligaments. But a sprain is more generally due to a lateral wrench, and then there is no bruising or abrasion of superficial structures. The pain and tenderness felt are referred to the lateral ligaments. It is important to know whether you are dealing with contusion due to direct violence, or with sprain due to indirect violence, because late complications may ensue in both conditions, and these vary with the cause.

After a severe wrench the examiner must take into consideration whether or not there are abnormal movements between the tibia and the femur. The abnormal mobility may take the form of lateral or antero-posterior displacement of the tibia on the femur. Such movements can occur only if there has been a rupture of the crucial ligaments, or fracture of the spine of the tibia; it is most important to recognise such an injury, because it may jeopardise the future integrity of the joint. This fracture can be demonstrated by radiography.

